# Five-year survival of class II restorations with and without base bulk-fill composite: a retrospective cohort study

**DOI:** 10.1007/s00784-024-05965-z

**Published:** 2024-09-30

**Authors:** Jukka Leinonen, Hannu Vähänikkilä, Remo Luksepp, Vuokko Anttonen

**Affiliations:** 1 Institute of Dentistry, School of Medicine, University of Eastern Finland, Kuopio, Finland; 2 Northern Finland Birth Cohorts, Arctic Biobank, Infrastructure for Population Studies, Faculty of Medicine, University of Oulu, Oulu, Finland; 3 Research Unit of Population Health, Faculty of Medicine, University of Oulu, Oulu, Finland

**Keywords:** General practice, Clinical, Dental restoration, Direct restoration, Longevity, Resin-based composite

## Abstract

**Objective:**

This study aimed to determine the survival of class II composite restorations in premolars and molars with and without base bulk-fill composite in general dental practice.

**Materials and methods:**

We collected data from the electronic patient files of the Public Dental Services in the City of Oulu, Finland. The timespan of data collection was from August 15th, 2002, to August 9th, 2018. The data consisted of class II composite restorations both with and without base bulk-fill composite. We compared the survival of these restorations using Kaplan–Meier survival curves, the log-rank test, survival rates, and the Wilcoxon signed ranks test.

**Results:**

We observed 297 restorations in 96 patients. The five-year survival rates for restorations with and without base bulk-fill composite were comparable in premolars (77.5% and 77.4%, respectively) but different in molars (69.9% and 57.8%, respectively, p = 0.069). In molars, the restorations with base bulk-fill composite exhibited a higher survival rate in 14 patients, whereas in 11 patients the restorations without base bulk-fill composites exhibited a higher survival rate. In 24 patients the survival rates were similar for restorations with and without the base bulk-fill composite (p = 0.246).

**Conclusions:**

The restorations with and without base bulk-fill composite had similar longevity.

**Clinical relevance:**

Base bulk-fill composites are safe to use in general practice due to their similar survival rates compared to conventional composites.

## Background

Dentists dedicate approximately 60% of their working hours to placing direct restorations [1]. These restorations are primarily replacement restorations and predominantly made of resin-based composite (hereafter referred to as “composite”) [2, 3]. Despite the widespread use of composite, it has several limitations, particularly related to longevity, time consumption, and technique sensitivity [4–6].

Conventional composites exhibit a paste-like consistency and can be applied in increments of up to two millimetres. In 2003, flowable base bulk-fill composites were introduced to reduce the number of steps and operation time required for composite restorative procedures [7]. Subsequently, paste-like bulk-fill composites became available. These bulk-fill composites achieve high polymerization degree even at the bottom of a four-millimetre increment [8]. Contrary to common belief, restorations with bulk-fill composites show minimal cuspal deflection and have good marginal integrity [9–11]. Additionally, bulk-fill composites exhibit good surface lustre and colour match [12, 13]. Notably, flowable base bulk-fill composites and conventional paste-like composites have nearly identical chemical-physical properties [14]. Due to the relatively low wear resistance of many flowable base bulk-fill composites, their instructions for use often recommend applying a more wear-resistant paste-like composite on top of the flowable base bulk-fill composite for occlusal surfaces [15]. Employing this “base bulk-fill technique” reduces the time needed to fill a large class II cavity by 60% or four and a half minutes compared to the conventional incremental technique [16]. But more importantly, the clinical performance of restorations with or without a bulk-fill composite is comparable in randomized controlled clinical trials [11, 12, 14, 17]. A meta-analysis comprising randomized controlled clinical trials has reported similar overall clinical success for restorations with and without bulk-fill composite [14]. Another meta-analysis comprising randomized controlled clinical trials has reported similar risks of retention/fracture, post-operative sensitivity, marginal adaptation, marginal discoloration, caries, anatomical form, surface texture and colour match for restorations with and without bulk-fill composite [17]. Two more recent randomized controlled trials have also reported similar clinical performance for restorations with and without bulk-fill composite [11, 12]. However, these randomized controlled trials have been performed in optimal conditions typical for randomized controlled trials: the dentists are select few specialists who follow the instructions for use in detail, use specific criteria to assess restorations, have good time to perform the restorative procedure and get to use their preferred instrumentation. Whereas in general practice many of these conditions are not optimal.

To the best of our knowledge, previous studies comparing the longevity of restorations with and without bulk-fill composites have been conducted under ideal conditions, addressing the question of efficacy: “Can bulk-fill composites work as well as conventional composites?” [18]. Our aim was to take the next step and address if the bulk-fill composites work as well as conventional composites under general practice settings [18]. Specifically, this retrospective controlled follow-up study aimed to compare the survival of class II composite restorations in premolars and molars with and without a base bulk-fill composite placed in general practice.

## Materials and methods

### Ethics

The study was conducted in accordance with the Declaration of Helsinki. Each individual participant does not need to provide their consent to participate for a retrospective data collection study such as this study. The study protocol was approved by the register holder, the City of Oulu Department of Healthcare (§9/2018 OUKA/973/07.01.04.02/2018).

### Data collection

We collected data on restorative procedures from patient records within the electronic practice management system (Effica®, Tieto, Helsinki, Finland) of the Public Dental Services in the City of Oulu, Finland. The inclusion criteria were that the patient had received class II composite restorations both with and without bulk-fill composite and had undergone a comprehensive oral examination at the City of Oulu Public Dental Services five years after the restorations had been placed. The latter criterion was used to minimize selection bias arising from patients relocating from Oulu or transitioning to a private dentist (whose records were inaccessible to us). We excluded patients under 18 years and patients over 60 years plus restorations covering both mesial and distal surfaces. The control restorations (without bulk-fill composite) were selected based on placement dates as close as possible to the date of the bulk-fill composite restoration placement. Data were collected for composite restorations placed before April 2013 to ensure a minimum five-year follow-up period by the end of the data collection in September 2018. The timespan of data collection was from August 15th, 2002, to August 9th, 2018.

For each restoration, we recorded the patient’s sex and date of birth, and operation date, restored tooth, restored surfaces, restorative material, operator, date when the restoration was assessed indicated for reoperation, the operator who performed the reoperation, and the indication for reoperation (secondary caries, chipping or lost restoration, access cavity preparation for endodontics, microleakage).

The working experience of the dentists was estimated by calculating the time from their graduation to the placement date of the restoration by subtracting the first of June of their graduation year from the date of the restoration placement. The graduation year of the dentist was found for 91.9% of the restorations [19].

### Statistics

Data were presented as frequencies, proportions, and means. The Wilcoxon signed ranks test was used to compare the rank of five-year survival for restorations with and without bulk-fill composite intra-individually. Separate Kaplan–Meier survival curves were drawn for restorations with and without bulk-fill composite for restorations in premolars and molars, in males and females, in 18–40-year-olds and 40–60-year-olds, in mandible and maxilla, on 1–2 surfaces and 3–4 surfaces, and in distal and mesial surfaces. Restorations that succeeded over five years were considered censored in the Kaplan–Meier analysis and registered with a survival time of five years. We assessed the statistical significance for the survival curves using the log-rank test. Furthermore, we conducted sensitivity analysis where we excluded stepwise premolars, male patients and restorations covering other than two surfaces. The mean annual failure rate at five years (AFR_5_) was determined using the formula: AFR_5_ = 1—$\sqrt[5]{x}$, where x represents the cumulative proportion surviving at five years. MedCalc (version 19.5.2., MedCalc Software Ltd, Ostend, Belgium) was used to plot the Kaplan–Meier survival curves. All other analyses were performed using SPSS (Statistical Package for the Social Sciences, version 29, IBM, Armonk, New York, USA). Results with p-value less than 0.05 were considered statistically significant.

## Results

### Demographics and distribution of restorations

Our data comprised 297 restorations placed by 50 dentists on 96 patients. Each patient had received one to six restorations with bulk-fill and one to five restorations without bulk-fill composite. The mean age of the patients at the time of restoration placement was 42.8 years (SD = 8.3 years). The only bulk-fill composite that had been used was the flowable base bulk-fill composite SDR (Dentsply Sirona, Charlotte, North Carolina, USA), which had been in use since February 2011. Just one of the restorations with SDR had only SDR whereas the rest had also Filtek Supreme XTE (46.0%, 3 M, St. Paul, MN, USA), Filtek Supreme (39.1%, 3 M), Filtek Supreme XT (13.7%, 3 M) or Grandio (0.6%, Voco, Cuxhaven, Germany). We assume that the Filtek Supremes and Grandio have been used to cover the occlusal surface as is instructed by the SDR instructions for use. The restorations without SDR had Filtek Supreme (30.9%), Filtek Supreme XTE (19.9%), Filtek Supreme XT (15.4%), Grandio (12.5%), Filtek Z100 (8.8%, 3 M), Filtek Z250 (4.4%, 3 M), Ceram.x (3.7%, Dentsply), Filtek (1.5%, 3 M), Filtek Flow (0.7%, 3 M), Admira (0.7%, Voco) and Prodigy (0.7%, Kerr, Brea, CA, USA). In the restorations with SDR the adhesives had been All-Bond 2 (0.6%, BISCO, Schaumburg, IL, USA), Clearfil SE Protect Bond (7.5%, Kuraray, Tokyo, Japan), Clearfil SE Bond (11.2%, Kuraray), Excite (0.6%, Ivoclar, Schaan, Liechtenstein), Scotchbond (3.7%, 3 M), Adper Scotchbond 1 XT (34.1%, 3 M) and Adper Scotchbond Multi-Purpose (16.1%, 3 M) whereas information about the adhesive used were not recorded for 26.1% of the restorations. In the restorations without SDR the adhesives had been AdheSE (0.7%, Ivoclar), Clearfil SE Protect Bond (0.7%), Clearfil SE Bond (8.1%), Excite (0.7%), Scotchbond (16.9%), Adper Scotchbond 1 XT (23.5%) and Adper Scotchbond Multi-Purpose (6.6%) whereas information about the adhesive used were not recorded for 41.9% of the restorations. The mean working experience of the dentists who had placed the restorations with bulk-fill composite was 17.1 years (SD = 8.0 years) and 14.8 years (SD = 8.6 years) for the dentists who had placed the restorations without bulk-fill composite.

Notably, 89.6% (266) of the restorations covered two surfaces, while 20 restorations covered three surfaces (occlusal surface, mesial or distal surface, and oral or buccal surface), 10 covered one surface, and one restoration extended to four surfaces (occlusal, mesial, buccal and lingual surfaces). Fifteen dentists had placed the 161 restorations using the base bulk-fill composite, whereas 49 dentists had placed the 136 restorations without the base bulk-fill composite. Twelve dentists had placed 8 to 34 restorations (78.1% of all restorations), while the remaining 38 dentists had placed fewer than five restorations. Patients had received two to ten restorations (mean 3.1, SD = 1.9). Notably, the dentist who placed and replaced the restoration were the same in only 14.7% of the cases.

### Survival rates of restorations

Out of the total, 206 restorations (69.4%) had survived for five years, resulting in a mean annual failure-rate of 7.1%. The distribution and survival rates of the restorations according to restorative material, tooth characteristics, and patient demographics are shown in Table 1.

**Table 1:** Distribution and survival for the class II composite restorations

Characteristics	Restorations, n (%)	Five-year survival rate, %	AFR_5_, %
Restoration material			
With bulk-fill composite	161 (54.2)	72.7	6.2
Without bulk-fill composite	136 (45.8)	65.4	8.1
Tooth type			
Premolar	111 (37.4)	77.4	5.0
Molar	186 (62.6)	64.5	8.4
Number of surfaces			
1	10 (3.4)	60.0	9.7
2	266 (89.6)	68.8	7.2
3–4	21 (7.0)	80.9	4.1
Restored surface			
Distal	161 (54.2)	70.2	6.8
Mesial	136 (45.8)	68.4	7.3
Jaw			
Mandibula	129 (43.4)	67.4	7.6
Maxilla	168 (56.6)	70.8	6.7
Patient gender			
Female	168 (56.6)	70.8	6.7
Male	129 (43.4)	67.4	7.6
Patient age			
18–40	212 (71.4)	70.3	6.8
40–60	85 (28.6)	65.1	8.2

**Fig. 1:**
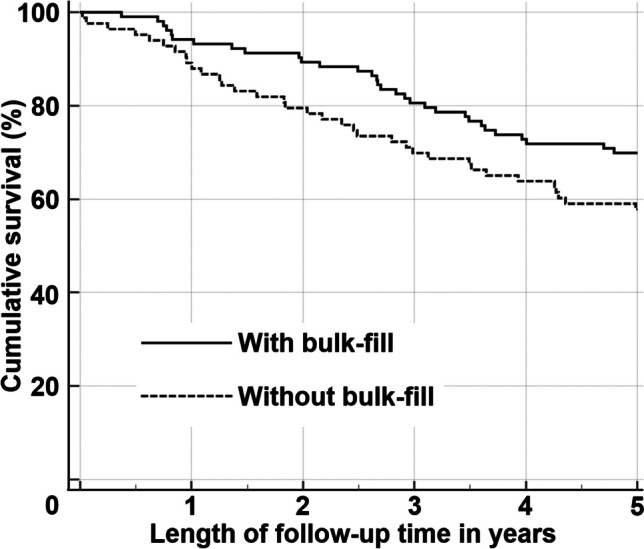
Kaplan–Meier survival curves for molar composite restorations with and without base bulk-fill composite (n = 186, p = 0.069)

The difference in five-year survival rates of molar restorations with and without bulk-fill composite was close to statistical significance (p = 0.069, Table 2, Fig. 1). Whereas the five-year survival rates for premolar restorations with and without bulk-fill were almost identical (Table 2). The distribution of restoration characteristics for restorations with and without bulk-fill composites were similar (Table 2).

**Table 2:** Five-year survival rate and number of restorations with and without base bulk-fill composite

Characteristics	With base bulk-fill composite (161)	Without base bulk-fill composite (136)	Statistical significance (log rank)
Tooth type			
Premolar	77.5% (58)	77.4% (53)	0.923
Molar	69.9% (103)	57.8% (83)	0.069
Number of surfaces			
1–2	71.3% (150)	65.1% (126)	0.195
3–4	90.9% (11)	70.0% (10)	0.268
Restored surface			
Distal	73.5% (83)	66.7% (78)	0.342
Mesial	71.8% (78)	63.8% (58)	0.232
Jaw			
Mandibula	68.8% (64)	66.2% (65)	0.774
Maxilla	75.3% (97)	64.8% (71)	0.930
Patient gender			
Female	78.7% (89)	67.0% (79)	0.086
Male	65.2% (72)	63.2% (57)	0.712
Patient age			
18–40	73.8% (107)	67.6% (105)	0.316
40–60	70.4% (54)	58.1% (31)	0.164

**Fig. 2:**
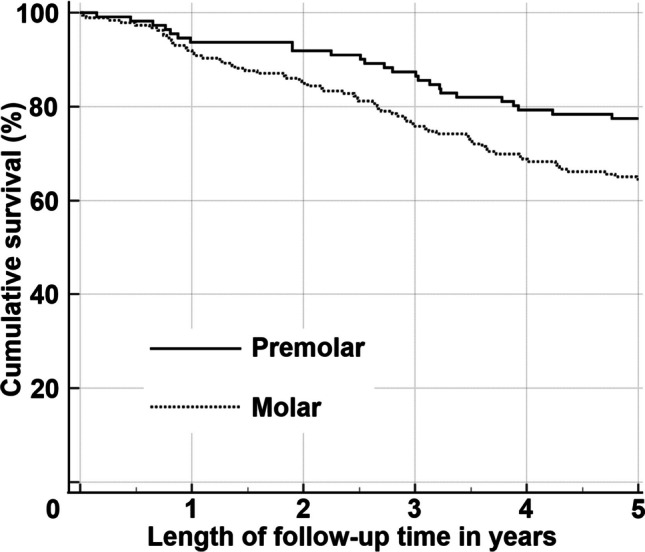
Kaplan–Meier survival curves for composite restorations in premolars and molars (n = 297, p = 0.019)

In 27 patients, the restorations with the bulk-fill composite exhibited a higher survival rate, whereas in 21 patients the restorations without the bulk-fill composite exhibited a higher survival rate. For 48 patients, the survival rates were comparable between restorations with and without the base bulk-fill composite (p = 0.466). When premolars were excluded from the analysis, the molar restorations with the bulk-fill composite exhibited a higher survival rate in 14 patients, whereas in 11 patients the molar restorations without the bulk-fill composite exhibited a higher survival rate. For 24 patients the survival rates were comparable between molar restorations with and without the base bulk-fill composite (p = 0.246). Notably, restorations in premolars demonstrated a superior five-year survival rate compared to restorations in molars (Fig. 2, p = 0.019). For the other characteristics in Tables 1 and 2, the differences in survival were not statistically significant. However, the sensitivity analysis revealed that the survival of restorations with base bulk-fill composite was higher than restorations without base bulk-fill composite in two-surface molar restorations that had been performed for female patients (p = 0.044, n = 89). The survival rate of restorations varied widely, ranging from 30.8% to 85.7% (mean 68.7%, SD = 13.4) for the twelve dentists who had placed more than seven restorations.

### Indications for reoperation

Indications for reoperation had been registered for 75.7% of the failed restorations. The distribution of the reoperation indications is shown in Table 3.

**Table 3:** The distribution of the reoperation indications (n = 136)

Indication for reoperation	With bulk-fill, n (%)	Without bulk-fill, n (%)
Chipping or lost restoration	23 (37.1)	19 (25.7)
Secondary caries	12 (19.4)	22 (29.7)
Access cavity preparation for endodontics	12 (19.4)	9 (12.2)
Microleakage	4 (6.5)	3 (4.1)
Not recorded	11 (17.7)	21 (28.4)

## Discussion

To the best of our knowledge, this is the first practice-based study on the longevity of bulk-fill materials. In premolars, the longevity for restorations with and without the base bulk-fill composite were nearly identical. Whereas in molars the class II restorations with base bulk-fill composite exhibited twelve percentage points better five-year survival than restorations without base bulk-fill composite – the difference was however not statistically significant. When we omitted men, premolar restorations and restorations covering other than two surfaces the association of base bulk-fill composite with longer survival of restorations was statistically significant. However, the post hoc selection of the omitted characteristics and the low number of restorations in the analysis prevent making conclusions from the sensitivity analysis. Our findings should alleviate some of the putative doubt surrounding the effectiveness of the bulk-fill base technique, even for those of us who have been educated in the oblique layering technique and might consider bulk-fill materials too good to be true.

Retrospective data collection studies, like ours, can identify correlations but not causal relations. A practice-based randomized controlled trial would be needed to conclusively determine if the base bulk-fill technique improves restoration longevity in general practice. That said, data collection studies and randomized controlled trials can complement each other. The randomized controlled trials comparing bulk-fill and conventional filling materials have not been performed in practice-based environments which might in part explain their low failure rate and equal longevity for restorations with and without bulk-fill composites [11, 12, 17]. The high survival rate of class II molar restorations with the base bulk-fill composite in our data prompts the question: “Does the base bulk-fill technique improve the longevity of class II molar restorations placed in general practice?”. However, recruiting general practitioners and patients for a practice-based randomized controlled trial is challenging and prone to selection bias of dentists whereas our data collection study included all dentists working for the Public Dental Services in the City of Oulu, Finland. Given the at least adequate longevity of restorations with bulk-fill composite, many dentists and patients probably already favour the bulk-fill composites due to their ease-of-use and time saving benefits. Thus, additional evidence on putative benefits on longevity by using bulk-fill technique from practice-based randomized controlled trials might not have a substantial effect on restorative technique or material preferences.

Unlike in general practice, operators in randomized controlled trials are often select few specialists who work under optimal clinical conditions. In optimal conditions posterior composite restorations, irrespective of the brand or type, have an excellent five-year survival rate of above 90% [20, 21]. Furthermore, in optimal conditions composite restorations in posterior teeth have a good long-term survival rate close to 60% in 27- to 30-year follow-ups [22, 23]. In general practice, the conditions are far from optimal because of e.g. time pressure and perhaps as a result, the five-year survival of class II composites is below 80% [24, 25]. The difference in the survival rates of restorations in randomized controlled trials and retrospective data collection studies could be partly explained by the conditions where the restoration has been placed but also by putative prevalent bruxism, high caries risk and hyposalivation (which are associated with short restoration longevity) or local threshold to initiate restoration replacement or repair [21, 26–28]. Interestingly, general practitioners expect their composite restorations to last less than seven years, which seems to be an underestimation [4, 24–26, 29]. Especially as one considers that it takes more than three years of gradual wearing before the composite restorations reach functional masticatory equilibrium [30].

In our data, secondary caries was a close second to chipping as an indication for reoperation. Secondary caries is the primary indication for restoration replacement in general practice, while few restorations fail due to secondary caries in randomized controlled trials [3, 11–13, 20, 21]. Surprisingly, adhesive strategies and restorative materials seem to play a lesser role in controlling secondary caries whereas patient- and operator-level factors seem decisive [31]. This might be a reason why so many randomized controlled trials have not detected a difference between survival of different filling materials – most filling materials perform well when they are being placed according to instructions for use and standard operating protocols plus assessed according to clear criteria [11–13, 20–23]. However, neither standard operating protocol nor clear filling assessment criteria were in use for the dentists in our data. Providing dentists with standard operating protocols for placing and assessing composite restorations plus enough time to work according to these protocols should be considered for means to improve the longevity of restorations. The operating protocols that have resulted in excellent longevity in prospective trials could be used for standard operating protocols.

Our data did not enable us to estimate how big a proportion of the restoration failures resulted from material, patient, and operator factors. However, our finding that the longevity of restorations varied greatly between dentists is in accord with previous studies [32–35]. The variation in the longevity between dentists may result in part from the patients assigned to dentists e.g., low socioeconomic status neighbourhood patients seeking dental care in their nearby clinic [36, 37]. However, since there is no standard operating protocol for the dentist working for the City of Oulu Public Dental Service, some of the variation in longevity of restorations between dentists may come from the chosen operating protocol. Perhaps because of time pressure the operating protocols (e.g. type of isolation and caries removal technique) had not been described in detail patient journals and thus could not be studied or described in this article. However, the restorations with and without base bulk-fill composite had been placed by dentists with similar working experience. In addition, the other filling materials, namely adhesives and conventional composites used, were similar for the restorations with and without base bulk-fill composite. Although most restorative materials have good longevity in randomized controlled trials, some restorative materials have shorter longevity than other materials in retrospective cohort studies [24, 35]. A putative explanation for the aforementioned finding (in addition to residual confounding) is that the restorative materials with short longevity are highly technique sensitive.

In our data the same dentist placed and evaluated the restoration for only 15% of the replacement restorations. This switching of dentists may explain some of the relatively short longevity of restorations found in our data [32, 38]. Furthermore, the patients in our data did might have received upon request restorations on teeth with a very poor prognosis (that would have been indicated for extraction) leading to subsequent restoration failure and further in part to the relatively short longevity of restorations in our data.

Restorations in molars had shorter longevity than those in premolars. Previous studies have identified a similar difference in the longevity of restorations in molars and premolars [25, 26, 34, 35]. The cause for the shorter longevity of molar restorations – whether due to technical challenges in placing the restoration, higher biting forces, difficulties performing oral hygiene, or other factors – remains elusive to the best of our knowledge. Considering that the premolar restorations with and without bulk-fill composite had nearly identical and adequate longevity in general practice, future studies aiming to demonstrate a positive effect on longevity when using bulk-fill composite could consider concentrating on molar teeth.

Public dental services are available to all Finnish citizens, with a third of adult Oulu habitants utilizing them at least once in 2017–2018 [39]. The heavy subsidizing of community dental charges in Finland assures high-quality in data collection studies as only 5% of patients visit both public dental services and private practitioners [39]. In Finland, patient records from private practitioners, are not available for retrospective data collection studies like ours. Our inclusion criterion of a comprehensive oral examination five years after placing the restorations ensured further that few if any patients had received dental care in a clinic whose records would have been unavailable to us. The treatment records in Finnish patient management systems adhere to strict national guidelines, ensuring their reliability [40].

The major strengths of this study are the practice-based setting, intra-individual comparison, long follow-up, and strict inclusion criteria. However, there are limitations in our study as well. Lack of randomization may have introduced selection bias regarding restorative materials. That is, in our data, the larger cavities with a poorer prognosis might have been predominantly chosen for the base bulk-fill technique due to its time-saving benefits especially in the large cavities [9, 16]. In a practice-based randomized controlled trial an equal number of large cavities would be filled using the base bulk-fill technique and the conventional incremental technique. Therefore, in a practice-based randomized controlled trial the difference in longevity of class II molar restorations with and without bulk-fill composite could be even larger than what we found. Another limitation to our study is that the data originated from the public dental services of one town, which could introduce biases related to the environment, operators, and patients. To minimize the patient-related bias, we compared data from the same patient for control and study group restorations. Due to our strict inclusion criteria, the sample size was too low for multivariable analyses. Also, some of the results that were close to statistically significant (at p < 0.05 level) might have been statistically significant if we had had a larger sample size. Finally, having 50 different dentists place the restorations introduced bias into the study.

## Conclusions

Class II molar restorations had shorter longevity than class II premolar restorations. The longevity of class II restorations with and without bulk-fill base composite was adequate and nearly identical in premolars. Whereas in molars the class II restorations with base bulk-fill composite exhibited twelve percentage points better five-year survival than restorations without base bulk-fill composite – the difference was however not statistically significant. A large-scale practice-based randomized control trial would be indicated to study if using bulk-fill composites improves the longevity of class II restorations in molars in general practice. However, base bulk-fill composites are safe to use in general practice due to their similar survival rates compared to conventional composites. Standard operating protocols for placing and assessing composite restorations plus enough time to work according to these protocols should be considered for means to improve the longevity of restorations.
